# miR-449a disturbs atherosclerotic plaque stability in streptozotocin and high-fat diet-induced diabetic mice by targeting CEACAM1

**DOI:** 10.1186/s13098-024-01322-y

**Published:** 2024-05-08

**Authors:** Jie Yu, Han Liu, Yu Chen, Ling Wang, Peng Chen, Yue Zhao, Chunxia Ou, Wei Chen, Jie Hu, Yu Wang, Yan Wang

**Affiliations:** 1 Department of Thoracocardiac Surgery, 920th Hospital of Joint Logistics Support Force of Chinese People’s Liberation Army, No.212 Daguan Rd, Kunming, Yunnan, 650032 China; 2 Department of Emergency Medicine, The First Affiliated Hospital of Kunming Medical University, No.295 Xichang Rd, Kunming, Yunnan 650032 China; 3 Laboratory of Molecular Cardiology, Department of Cardiology, The First Affiliated Hospital of Kunming Medical University, No.295 Xichang Rd, Kunming, Yunnan 650032 China; 4 Department of Cardiology, 920th Hospital of Joint Logistics Support Force of Chinese People’s Liberation Army, No.212 Daguan Rd, Kunming, Yunnan, 650032 China; 5 Department of Radiology, The First Affiliated Hospital of Kunming Medical University, No.295 Xichang Rd, Kunming, Yunnan 650032 China

**Keywords:** MiR-449a, Plaque stability, Diabetes mellitus, Atherosclerosis, CEACAM1

## Abstract

**Background:**

Emerging evidence indicates carcinoembryonic antigen-related cell adhesion molecule 1 (CEACAM1) is involved in the development of atherosclerosis (AS). However, the roles and functions of CEACAM1 in AS remain unknown. Therefore, this study aims to investigate the roles and molecular functions of CEACAM1 in AS.

**Methods:**

We constructed a diabetes mellitus (DM) + high-fat diet (HFD) mouse model based on the streptozotocin (STZ)-induced apolipoprotein E-knockdown (ApopE^−/−^) mouse to investigate the roles and regulatory mechanism of miR-449a/CEACAM1 axis. The mRNA expression and protein levels in this study were examined using quantity PCR, western blot, immunofluorescence (IF), enzyme-linked immunosorbent assay (ELISA), and immunohistochemistry (IHC), respectively. And the lipid deposition and collagen content were detected using Oil Red O and Sirius Red staining. Cell apoptosis, migration, invasion, and tuber formation were detected by Annexin-V FITC/PI, wound healing, transwell, and tuber formation assays, respectively. The relationship between miR-449a and CEACAM1 was determined by a dual-luciferase reporter gene assay.

**Results:**

miR-449a and MMP-9 were upregulated, and CEACAM1 was downregulated in the DM + HFD MOUSE model. Upregulation of CEACAM1 promoted atherosclerotic plaque stability and inhibited inflammation in the DM + HFD mouse model. And miR-449a directly targeted CEACAM1. Besides, miR-449a interacted with CEACAM1 to regulate atherosclerotic plaque stability and inflammation in DM-associated AS mice. In vitro, the rescue experiments showed miR-449a interacted with CEACAM1 to affect apoptosis, migration, invasion, and tuber formation ability in high glucose (HG)-induced HUVECs.

**Conclusion:**

These results demonstrated that miR-449a promoted plaque instability and inflammation in DM and HFD-induced mice by targeting CEACAM1.

**Supplementary Information:**

The online version contains supplementary material available at 10.1186/s13098-024-01322-y.

## Introduction

Diabetes mellitus is a globally epidemic disease, which includes 415 million diagnosed and 193 million undiagnosed diabetes worldwide in 2015, and more than 90% of those patients have been accounted for as type 2 diabetes mellitus (T2DM) [1]. Complications of atherosclerosis (AS) are the leading cause of rises in morbidity and mortality in diabetes mellitus (DM) patients [2–4]. The autoimmune destruction of pancreatic β-cells causes type 1 diabetes mellitus (T1DM), but T2DM is non-insulin-dependent diabetes [5], which primarily results from the interaction of genetic, environmental, and other risk factors [6]. AS has been characterized by arterial wall thickening and lipid accumulation promoting plaque formation; the process of AS includes initiation, progression, and plaque disruption [7]. During the AS processing, several types of cells are involved in this process, such as vascular endothelial cells (VECs), leukocytes (e.g., T cells, macrophages, etc.), and vascular smooth muscle cells (VSMCs) [8]. VECs serve as a plate for molecules to adhere to and permit their penetration underneath the vascular layer to initiate the inflammatory process. Then, infiltrated macrophages engulf the oxidized low-density lipoprotein (ox-LDL), convert to large foam cells, and then die, aggravating the inflammation process by inducing hypoxic, necrotic, and pro-inflammatory core formation. Known from the early events of inflammation and inflammatory core formation, VSMCs respond to inflammation by producing collagen and then forming a fibrous cap against plaque rupture and thrombosis [8, 9]. Macrovascular complications, including AS, are major causes of morbidity and mortality in T2DM patients [10]. Although knowledge of the pathology of AS and exploration of abundantly regulatory genes over the past decades, the outcome of DM-associated AS remains unsatisfactory.

Carcinoembryonic antigen-related cell adhesion molecule 1 (CEACAM1) is a member of the immunoglobulin superfamily that functions as an adhere molecule and modulates vascular permeability [11]. CEACAM1 contributes to aging-related vascular disorders such as endothelial dysfunction and atherosclerotic plaque formation via collagen depositions [12]. CEACAM1 plays a dominant role in vascular homeostasis by regulating endothelial function and inflammation response in AS and cardiovascular dysfunction [13–15]. Despite the fact that we have proved the function and mechanism of CEACAM1 in the diabetic AS in the previous study [22]. However, the upstream regulatory mechanism remains unclear. Increasing evidence highlights that microRNAs (miRNAs) play essential roles in the initiation, development, and plaque rupture by directly interacting with target genes in regulating transcriptional and post-transcriptional gene expression [16, 17]. Only knowledge on miR-342 and miR-30b/30d targeting CEACAM1 in the lumen formation of breast epithelium and fulminant hepatic failure (FHF) [18, 19]. The abundant number of miRNAs targeting CEACAM1 remains unfound. Previous studies have found that miR-449a is essential in the development of atherosclerosis [20, 21]. Nevertheless, whether miR-449a interacts with CEACAM1 to affect atherosclerosis progression remains unknown.

Here, we investigated the role of CEACAM1 and the regulatory mechanism of the miR-449a/CEACAM1 axis in DM-associated AS. We constructed the DM- and HFD-induced mice and HG-induced cell models to investigate the role and mechanism of the miR-449a and CEACAM1 in DM-associated AS. We found miR-449a promotes plaque rupture via targeting CEACAM1 in DM- and HFD-induced mice.

## Methods

### Animals

All animal experiments in this study were approved by the Ethics Committee of Yunnan Labreal Biotech Ltd. Co., and operations followed the standard operating procedures of the Laboratory Animal Center of Kunming Medical University (No. PZ20230109). A total of 40 eight-week-old male apolipoprotein (Apo E^−/−^) mice (22.0 ± 0.4 g) were provided by the Animal Center of Kunming Medical University. Firstly, DM mice were established via daily injecting with 50 mg/kg streptozotocin (STZ; Sigma-Aldrich, MO, USA) for five days [22]. The control group mice were accepted with an equal volume of citric acid (CA) for five consecutive days. The DM mice were confirmed with blood glucose > 16.7 mM then were randomly distributed into seven groups including DM + HFD group (*n* = 5), DM + HFD + pcDNA3.1 group (*n* = 5), DM + HFD + CEACAM1 group (*n* = 5), DM + HFD + NC inhibitor group (*n* = 5), DM + HFD + miR-449a inhibitor group (*n* = 5), DM + HFD + miR-449a inhibitor + Si-NC group (*n* = 5), DM + HFD + miR-449a inhibitor + Si-CECAM1 group (*n* = 5). Subsequently, the DM mice were fed a high-fat diet (HFD), including 0.25% cholesterol and 20% lard oil, for four weeks to establish the AS mouse model. The control mice were fed with standard mice chow. All mice were grown in living conditions with 18–22°C, 50% humidity, and a 12 h light/dark cycle. The formation of atherosclerotic plaque was confirmed with transabdominal aortic ultrasound, and the DM mice with an atherosclerotic plaque served as DM-induced AS mice. After four weeks, the DM + HFD mice were injected with lentivirus expressing CEACAM1 (2 × 10^7^ TU/each mouse) or lentivirus expressing NC (2 × 10^7^ TU/each mouse) by tail vein injection or miR-449a inhibitor and its negative control, or si-CECAM1 and its negative control. The control and DM + HFD group mice were injected with equal saline and then fed with standard mice chow for another sixteen weeks. At the end of the twentieth week, all mice were euthanized with excessive inhalation of isoflurane, and then blood samples and the thoracic aortic tissues were harvested from subsequent experiments. The body weight of each mouse was calculated, and the biochemical measurements, including oxidized low-density lipoprotein (Ox-LDL), T-CHO (total cholesterol), total triglycerides (TG), and blood glucose (BG), were measured. A flow chart of animals interfering in this study is shown in Supplementary Fig. 1.

### Immunofluorescence (IF)

After the euthanasia, the aorta roots were separated from the mice and immediately fixed with 4% paraformaldehyde (PFA). The sections were paraffin-embedded and subjected to pathological and immunological staining. To investigate the levels of CEACAM1 on the surface of the aorta using the IF staining. The paraffin-embedded sections were sliced into the 4-µm thickness and dehydrated with xylene and gradient ethanol. The slices were incubated with primary CEACAM1 monoclonal Antibody (MA5-24338; Thermo Fisher Scientific, Waltham, MA, USA) at 25 µg/mL overnight at 4°C. After that, the sections were incubated with Goat Anti-Rat IgG NorthernLights™ NL557-conjugated Antibody (1:200, NL013; R&D Systems, Minneapolis, MN, USA) for 2 h at room temperature in the dark. The nuclear was stained with 4’, 6‑diamidino‑2‑phenylindole (DAPI; Sigma-Aldrich, St. Louis, MO, USA) for 5 min. After washing the sections three times, the positive cells were observed and photographed using a confocal fluorescence microscope (UltraView Vox; PerkinElmer, MA, USA).

### Histological staining and immunochemistry (IHC)

The aortic sinus lesions were detected using an Oil Red O staining kit (Beyotime, Shanghai, China) and Sirius Red Sigma-Aldrich (365548-25G; St. Louis, MO, USA, respectively. Oil Red O staining was used to examine the lipidosis. Briefly, the 4-µm sections were stained with Oil Red O staining solution for 20 min at room temperature according to the manufacturer’s instruction, then rinsed with phosphate buffer saline (PBS) three times, then lipid deposition was observed and imaged under an inverse microscope (Leica, Wetzlar, Germany). Additionally, Sirius Red staining was used to detect the interstitial collagen content of the atherosclerotic plaques. The sections were stained with Sirius red staining solution and incubated at room temperature for 30 min. The collagen of the plaques was observed and photographed under an inverse microscope (Leica, Wetzlar, Germany). In addition, the numbers of VSMCs and macrophages were examined using IHC staining with SMCs marker α smooth muscle actin (a-SMA, 1:1000; ab124964, Abcam, Cambridge, MA, USA) and macrophage marker (MOMA2, 1:500; ab33451, Abcam, Cambridge, MA, USA). Then, the plaque vulnerable index was then calculated according to the ratio between the Oil red O positive area plus the MOMA-2 positive area and the Sirius Red area plus a-SMA area. Moreover, the levels of VECs marker CD34 (1:2500; ab82289, Abcam, Cambridge, MA, USA) and vascular endothelial growth factor (VEGF, 1:100; MA5-13182, Thermo Fisher Scientific, Waltham, MA, USA) were detected with IHC staining. In brief, 4-µm sections were incubated with primary antibodies overnight at 4°C and stained with Goat anti-rabbit IgG HRP-conjugated secondary antibody (1:1000; ab150077, Abcam, Cambridge, MA, USA) at room temperature for 1 h. Finally, the sections were counterstained with hematoxylin for 1 min and blued with 1% ammonia water. The positive rate was measured by the product of the percentage of immunopositive area (0%, 0; 1–25%, 1; 26–50%, 2; 51–75%, 3; 76–100%, 4) and staining intensity (0, negative; 1, weak positive; 2, medium positive; 3, strong positive). The images were measured using Image J software.

### Enzyme-linked immunosorbent assay (ELISA)

The levels of inflammatory factors tumor necrosis factor-α (TNF-α), interleukin-1β (IL-1β), interleukin-6 (IL-6), and interleukin-8 (IL-8) were measured using the ELISA kits (R&D Systems, Minneapolis, MN, USA) following the manufacturer’s suggestion. The expression of adhesion molecules such as vascular cell adhesion molecule 1 (VCAM-1), intercellular adhesion molecule 1 (ICAM-1), and macrophage cationic peptide 1 (MCP-1) was also examined using ELISA kits (Abcam, Cambridge, MA, USA).

### Apoptosis analysis in vivo

TUNEL staining was used to determine the apoptotic rate of the VECs in vivo. In brief, the atherosclerotic plaque sections were stained with anti-CD34 antibody overnight at 4°C. Subsequently, the sections were stained with TUNEL staining solution (Beyotime, Shanghai, China) following the manufacturer’s protocol. After that, the sections were stained with Goat anti-rabbit IgG HRP-conjugated secondary antibody as previously described. Finally, the apoptotic VECs were calculated according to the total of the CD34 and TUNEL positive cells relative to the total number of the CD34 positive cells in the plaque.

### Cell culture and transfection

Human umbilical vein endothelial cells (HUVECs) were purchased from the American Type Culture Collection (ATCC, Manassas, VA, USA). HUVECs were cultured in the EGM-2 medium (Lonza, Basel, Switzerland). Then, HUVECs were incubated in a humid incubator with 37°C and 5% CO_2_. HUVECs were seeded into six-well plates at 4 × 105 cells/well density and cultured at 37°C with 5% CO_2_ until cells reached 80–90% confluence. HUVECs were stimulated with a high glucose (33 mM) medium for five days in the absence or presence of miRNA inhibitors and si-RNAs. Then, HUVECs were randomly divided into five groups, including the HG group, HG + NC inhibitor group, HG + miR-449a inhibitor group, HG + miR-449a inhibitor + Si-NC group, HG + miR-449a inhibitor + Si-CEACAM1 group. Exception of the control group, miRNA inhibitor, and interfering RNA subsequently transfected into HUVECs using Lipofectamine 3000 regents (Invitrogen, Carlsbad, CA, USA) following the manufacturer’s instruction.

### Apoptosis analysis in vitro

The apoptosis was detected using the Annexin V-FITC/PI detection kit (Beyotime, Shanghai, China) according to the manufacturer’s suggestion. Briefly, the transfected-HUVECs were collected and resuspended in 500 µL PBS at a density of 1 × 10^5^ cells. Then, cells were centrifuged at 1000 g for 5 min and resuspended with 195 µL Annexin V-FITC binding solution. After that, 5 µL Annexin V-FITC and 10 µL Propidium iodide (PI) staining solution were added to cells and then incubated for 20 min at room temperature in the dark. Finally, apoptotic cells were detected using flow cytometry (FCM).

### RNA extraction and quantitative PCR (qPCR)

Total RNA and microRNA were extracted from tissues and cells using Trizol reagent (Takara, Dalian, China) and RNAiso for Small RNA (Takara, Dalian, China) following the manufacturer’s suggestion, respectively. RNA was reversed transcription into cDNA using PrimeScript™ RT reagent Kit (Takara, Dalian, China) according to the manufacturer’s protocol. Then, cDNA was performed qPCR analysis with TB Green® Fast qPCR Mix (Takara, Dalian, China) on an ABI Prism 7300 system (Applied Biosystems, Waltham, MA, USA). The amplification conditions include 95°C for 15 s, 95°C for 5 s, and 60°C for 30 for 42 cycles. The relative expression of genes was determined using 2^−ΔΔCt^ methods, which calculated ΔCt = Ct (target gene) – Ct (reference gene) and ΔΔCt = ΔCt (experimental group) - ΔCt (control group). GAPDH and U6 were used to normalize gene expression. The primers were listed as follows, miR-449a, F, 5’-GTGTGATGAGCTGGCAGTGTA-3’, R, 5’-AGCAGTTGCATGTTAGCCGAT-3’. CEACAM1, F, 5’-GCTGGGACGTATTGGTGTGA-3’, R, 5’-GTCATTGGAGTGGTCCTGCC-3’. MMP-9, F, 5’-TCTATGGTCCTCGCCCTGAA-3’, R, 5’-TTGTATCCGGCAAACTGGCT-3’. U6, F, 5’-GGCTCAGAATCACCCCATGT-3’, R, 5’-CCTGGACGTGCAGATGACTT-3’. GAPDH, F, 5’-GGTCACCAGGGCTGCTTTTA-3’, R, 5’-CCCGTTCTCAGCCATGTAGT-3’.

### Dual-luciferase reporter gene assay

The sequences of 3’UTR of CEACAM1 contained binding sites (CACUGCC) with miR-449a were amplified and inserted into luciferase reporter plasmids to construct the wild-type recombinational luciferase reporter genes (CEACAM1-WT). In addition, the binding site sequences were mutated into CUGACGC, and the mutant-type recombinational luciferase reporter genes (CEACAM1-MUT) were constructed. Then, the recombinational wild/mutant type luciferase reporters were con-transfected with miR-499a mimic or its negative control into HUVECs and incubated for 48 h. After that, the luciferase activity was measured using a dual-luciferase activity reporter system (Promega, Madison, WI, USA) according to the manufacturer’s protocol.

### Western blot assay

Protein was isolated from tissues and cells using Cell lysis buffers (Takara, Dalian, China) following the suggestion of the manufacturer. Subsequently, the protein was separated by 10% SDS-PAGE gel, transferred with PVDF membranes, and blocked with 5% non-fat milk. After that, the membranes were incubated with rabbit anti-MMP9 antibody (1:1000; ab38898, Abcam, Cambridge, MA, USA), rabbit anti-TIMP1 antibody (1:1000; ab216432, Abcam, Cambridge, MA, USA), rabbit anti-CEACAM1 antibody (1:1000; #14,771, Cell Signaling Technology, Danvers, MA, USA), rabbit anti-GAPDH antibody (1:2000; ab8245, Abcam, Cambridge, MA, USA) overnight at 4°C. The membranes were further incubated with HRP-labeled goat anti-rabbit IgG (1:2000; ab7090, Abcam, Cambridge, MA, USA) at room temperature for 1 h. Finally, bands were visualized using an enhanced chemiluminescence reaction solution (Bio-Rad, Hercules, CA, USA). GAPDH is used as an internal reference gene to normalize protein levels.

### Migration and invasion assay

The cell migration and invasion were examined using the wound healing and Transwell assays. After HUVECs transfection or stimulation, cells were harvested and seeded into the six-well plates at the density of 1 × 10^6^ cells/well, then incubated overnight at 37°C. Subsequently, the monolayer cells were scratched using a 200 µL pipette. The migrated areas of the migrated cells were observed and photographed at 0 h and 24 h. In addition, the transfected and stimulated HUVECs (2 × 10^5^ cells/well) were collected and seeded onto the transwell insert, which was pro-coated with Matrigel (BD Biosciences, San Jose, CA, USA). 500 µL EGM-2 medium was added into the lower well and incubated for 24 h. Finally, the invaded cells on the opposite filters were fixed with methanol and stained with 1% crystal violet (Beyotime, Shanghai, China) according to the manufacturer’s protocol. And the invaded cells were observed and counted under an inverted microscope (Leica, Wetzlar, Germany).

### Tube formation assay

The tube formation was performed using Matrigel (BD Biosciences, San Jose, CA, USA). After cell transfection and stimulation, 1 × 10^4^ HUVECs were seeded onto the Matrigel pre-coated 96-well plates and incubated in the EGM-2 medium for 12 h. The tube formation of HUVECs was visualized, and the number of nodes was calculated according to at least three cells formed at a single point quantitated as a node.

### Statistical analysis

All values in this study were expressed as mean ± stand deviation (SD). Comparison between multiple groups was performed using one/two-way analysis of variance (ANOVA), respectively. All statistical analyses were performed using GraphPad Prism 9 (GraphPad, Inc).

## Results

### Downregulation of CEACAM1 in STZ and HFD-induced diabetic mice

**Fig. 1:**
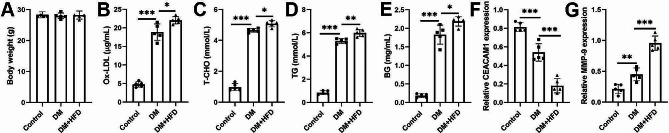
Downregulation of CEACAM1 in STZ and HFD-induced diabetic mice. (**A**) Body weight of the STZ-induced diabetic mice with and without HFD, and control mice. (**B**)-(**E**) Biochemical measurement of the serum levels of low-density lipoprotein (Ox-LDL), T-CHO (total cholesterol), total triglycerides (TG), and blood glucose (BG) of STZ-induced diabetic mice with and without HFD, and control mice. (**F**)-(**G**) QPCR analysis of the CEACAM1 and MMP-9 expression in STZ-induced diabetic mice with and without HFD, and control mice. The Scatter plot represented values obtained from five individual mice, and the significances between those groups were obtained by the value (mean ± SD) analyzed by one-way ANOVA. *P < 0.05; **P < 0.01; ***P < 0.001

Firstly, we tested whether CEACAM1 was abnormally expressed in STZ and HFD-induced diabetic mice. Mice were treated according to the description in Figure S1. We found there are no significant differences in body weight of the STZ-induced alone, and the STZ and HFD-induced diabetic mice than control mice (Fig. 1A). However, the levels of Ox-LDL, T-CHO, TG, and BG increased both in STZ-induced alone and in the STZ- and HFD-induced diabetic mice compared with control mice (Fig. 1B-E). Additionally, we found the expression of CEACAM1 was upregulated, whereas MMP-9 was downregulated in both STZ-induced alone and the STZ- and HFD-induced diabetic mice compared with control mice (Fig. 1F-G). These data suggested that CEACAM1 exerted an essential role in diabetes.

### Upregulation of CEACAM1 promotes atherosclerotic plaque stability in STZ and HFD-induced diabetic mice

**Fig. 2:**
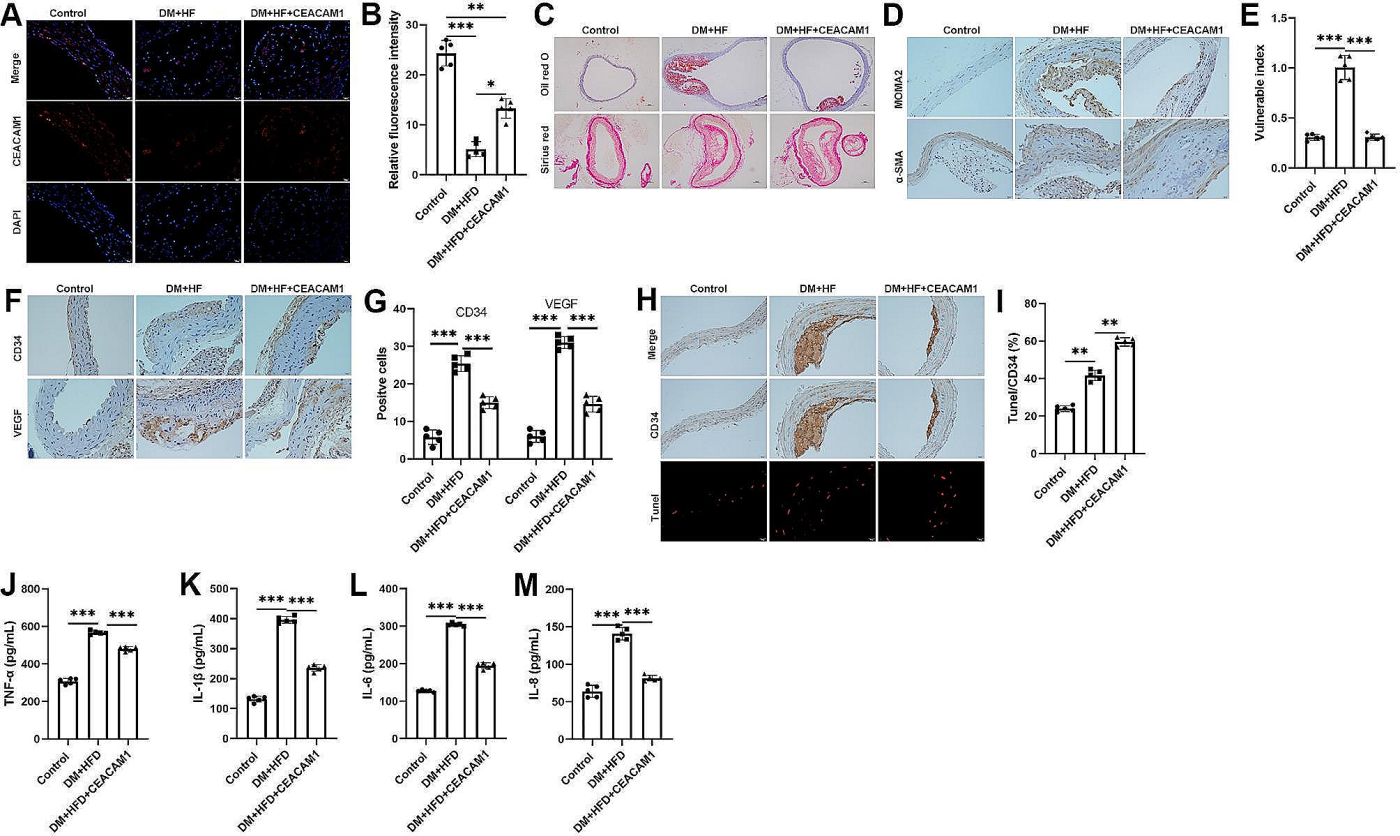
Upregulation of CEACAM1 promotes atherosclerotic plaque stability in STZ and HFD-induced diabetic mice. (**A**)-(**B**) Immunofluorescence analysis of the levels of CEACAM1 in STZ and HFD-induced DM mice after being subjected to CEACAM1 overexpression. (**C**) The histological analysis was performed by Oil red O and Sirius red staining in STZ and HFD-induced DM mice after being subjected to CEACAM1 overexpression. (**D**) Immunohistochemistry analysis of the levels of MOMA-2 and α-SMA in STZ and HFD-induced DM mice after being subjected to CEACAM1 overexpression. (**E**) The plaque vulnerable index was calculated according to the formula: vulnerable index = (Oil red O positive area + MOMA-2 positive area) / (Sirius Red area + a-SMA area). (**F**)-(**G**) Immunohistochemistry analysis of the levels of CD34 and VEGF in STZ and HFD-induced DM mice after being subjected to CEACAM1 overexpression. (**H**)-(**I**) Immunohistochemistry and TUNEL assay detected the apoptotic VECs in STZ and HFD-induced DM mice after being subjected to CEACAM1 overexpression. (**J**)-(**M**) ELISA detected the levels of TNF-α, IL-1β, IL-6, and IL-8 in STZ and HFD-induced DM mice after being subjected to CEACAM1 overexpression. The Scatter plot indicated values obtained from five individual mice, and the significances between those groups were obtained by the value (mean ± SD) were analyzed by one-way ANOVA. *P < 0.05; **P < 0.01; ***P < 0.001

CEACAM1 expression involves in vascular homeostasis in atherosclerosis [12]. While the function of the CEACAM1 in diabetes facilitates plaque formation remains not entirely understood. Therefore, we next investigated whether CEACAM1 affects plaque stability and inflammation in STZ and HFD-induced diabetic mice. Here, the STZ and HFD-induced ApoE^−/−^ mouse model was developed to explore the function of CEACAM1 in vivo. As shown in Fig. 2A, the expression of CEACAM1 decreased in the STZ and HFD-induced mice, whereas the CEACAM1 was stably expressed by using the lentivirus. In addition, we examined the plaque stability according to the vulnerable index. The results indicated that overexpression of CEACAM1 repressed the lipid deposition and macrophages increasing, but increased the collagen content and the number of VSMCs (Fig. 2C-D). According to the calculation of the vulnerability index, we found that the upregulation of CEACAM1 promotes the stability of atherosclerotic plaque in STZ and HFD-induced diabetic mice. Moreover, the IHC results revealed that an increasing number of VECs in STZ and HFD-induced diabetic mice were repressed by CEACAM1 upregulation (Fig. 2F-G). We also observed that CEACAM1 upregulation notably induced the apoptosis of ECs (Fig. 2H-I). Besides, the levels of inflammatory factors IL-1β, IL-6, IL-8, and TNF-α were remarkably increased in STZ and HFD-induced diabetic mice, whereas they were reduced by CEACAM1 upregulation (Fig. 2J-M). To sum up, our results indicated that the upregulation of CEACAM1 contributed to plaque stability in STZ and HFD-induced diabetic mice.

### CEACAM1 is a target of miR-449a

**Fig. 3:**
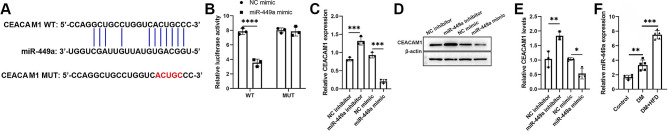
CEACAM1 is a target of miR-449a. (**A**) The binding sites between miR-449a and CEACAM1 were predicated using Starbase online database. (**B**) Dual-luciferase activity analysis after miR-449a mimic co-transfected with wild type 3’-UTR CEACAM1 recombination reporter gene or mutant type 3’-UTR CEACAM1 recombination reporter gene. (**C**)-(**E**) QPCR and western blot analysis of the levels of CEACAM1 by CEACAM1 silencing and overexpressing. (**F**) QPCR analysis of miR-449a expression in STZ-induced diabetic mice with and without HFD. Scatter plots represented values obtained from three individual experiments or five individual mice, and the significances between those groups were obtained by the value (mean ± SD) were analyzed by one/two-way ANOVA. *P < 0.05; **P < 0.01; ***P < 0.001

We further examine the upstream regulatory mechanism of CEACAM1 in STZ and HFD-induced diabetic mice. miRNAs have emerged as a major player in various diseases. Therefore, we focused on the miRNAs that interacted with CEACAM1. According to the Starbase online database (http://starbase.sysu.edu.cn/index.php), we found CEACAM1 is a target of miR-449a; the speculated binding sites between miR-449a and CEACAM1 were shown in Fig. 3A. The speculated relationship was determined by a dual-luciferase activity assay; we found that the CEACAM1-WT recombined reporter gene co-transfected with miR-449a mimics strongly inhibited luciferase activity whereas there was no change by CEACAM1-MUT recombined reporter gene co-transfected with miR-449a mimic (Fig. 3B). qPCR and western blot indicated the mRNA and proteins of CEACAM1 were upregulated by miR-449a inhibition, whereas they were repressed by miR-449a overexpression (Fig. 3C-E). We also detected the expression of miR-449a in the STZ and HFD-induced diabetic mice; the results illustrated that it was upregulated both in STZ and HFD-induced diabetic mice (Fig. 3F).

### MiR-449a aggravates plaque rapture via inhibiting CEACAM1 in STZ and HFD-induced diabetic mice

**Fig. 4:**
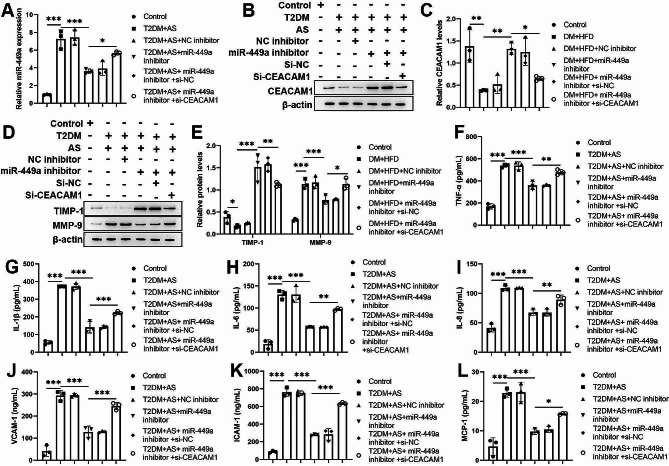
MiR-449a promotes inflammation by targeting CEACAM1. (**A**) QPCR analysis of miR-449a expression in STZ and HFD-induced DM mice after transfected with miRNA inhibitors and small interfering RNAs. (**B**)-(**E**) Western blot analysis of the levels of CEACAM1, TIMP-1, and MMP-9 in STZ and HFD-induced DM mice after being transfected with miRNA inhibitors and small interfering RNAs. (**F**)-(**L**) ELISA analysis of the inflammation cytokines (TNF-α, IL-1β, IL-6, IL-8) and adhere molecules (VCAM-1, ICAM-1, MCP-1) in STZ and HFD-induced DM mice after transfected miRNA inhibitors and small interfering RNAs. The Scatter plot represented values obtained from five individual mice, and the significances between those groups were obtained by the value (mean ± SD) analyzed by one-way ANOVA. *P < 0.05; **P < 0.01; ***P < 0.001

**Fig. 5:**
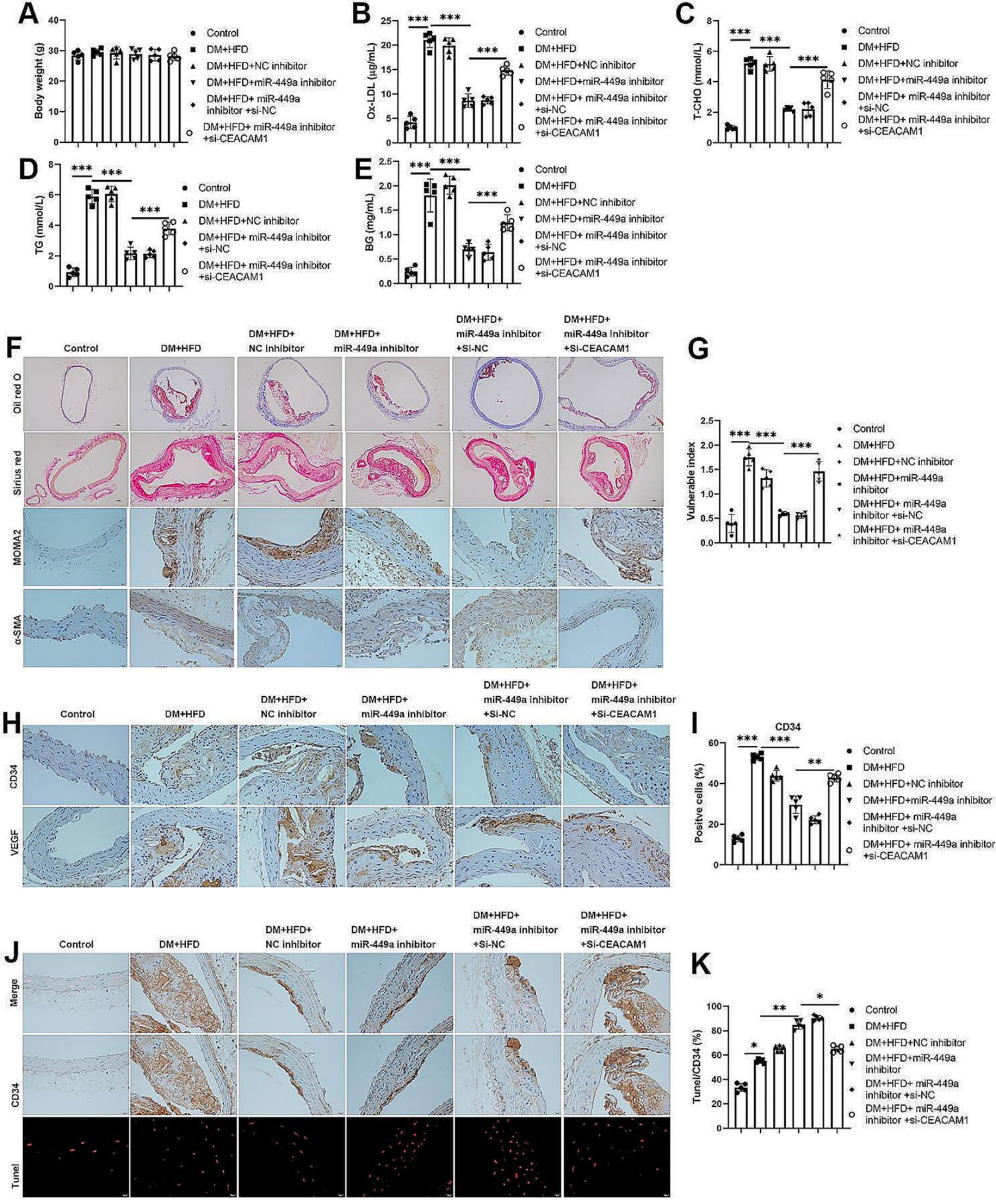
MiR-449a aggravates plaque rapture via inhibiting CEACAM1 in STZ and HFD-induced diabetic mice. (**A**) Body weight of the STZ and HFD-induced DM mice after being transfected with miRNA inhibitors and small interfering RNAs. (**B**)-(**E**) Biochemical measurement of the serum levels of Ox-LDL, T-CHO, TG, and BG of the STZ and HFD-induced DM mice after being transfected with miRNA inhibitors and small interfering RNAs. (**F**) The histological analysis was performed by Oil red O and Sirius red staining, and immunohistochemistry analysis of the levels of MOMA-2 and α-SMA in STZ and HFD-induced DM mice after transfected with miRNA inhibitors and small interfering RNAs. (**G**) The plaque vulnerable index was calculated according to the formula: vulnerable index = (Oil red O positive area + MOMA-2 positive area) / (Sirius Red area + a-SMA area). (**H**)-(**I**) Immunohistochemistry analysis of the levels of CD34 and VEGF in STZ and HFD-induced DM mice after transfected with miRNA inhibitors and small interfering RNAs. (**J**)-(**K**) Immunohistochemistry and TUNEL assay detected the apoptotic VECs in STZ and HFD-induced DM mice after being transfected with miRNA inhibitors and small interfering RNAs. The Scatter plot represented values obtained from five individual mice, and the significances between those groups were obtained by the value (mean ± SD) analyzed by one-way ANOVA. The data for Control and DM + HFD are shared in Fig. 1. *P < 0.05; **P < 0.01; ***P < 0.001

We further investigate the regulatory mechanism of the miR-449a/CEACAM1 axis in vivo. qPCR results demonstrated miR-449a upregulated in STZ and HFD-induced diabetic mice, and the expression of miR-449a was inhibited by miR-449a inhibitor. However, the effects of miR-449a inhibitor were neutralized by CEACAM1 knockdown (Fig. 4A). Western blotting indicated protein levels of CEACAM1 and TIMP-1 were reduced in STZ and HFD-induced diabetic mice; knockdown of miR-449a increased CEACAM1 and TIMP-1 levels and subsequently reduced by knockdown of CEACAM1; however, MMP-9 exhibited the opposite expression by the previous interfering (Fig. 4B-E). ELISA results showed that the levels of TNF-α, IL-1β, IL-6, IL-8, VCAM-1, ICAM-1, and MCP-1 were significantly upregulated in STZ and HFD-induced diabetic mice, and these levels were reduced by miR-449a inhibition but reversed by CEACAM1 knockdown (Fig. 4F-L). We also found that the mice’s body weight was not significantly different in different treatment groups (Fig. 5A). Further, the knockdown of miR-449a reduced the oxidized low-density lipoprotein, total cholesterol, total triglycerides, and blood glucose in STZ and HFD-induced diabetic mice, but the knockdown of CEACAM1 reversed those phenomena (Fig. 5B-E). However, the histological and IHC staining showed inhibited lipid deposition and macrophages. Still, the collagen content and number of VSMCs were increased by miR-449a inhibition, but the effects of miR-449a inhibitor were partly reversed by CEACAM1 knockdown (Fig. 5F). Statistically, the vulnerability index of STZ and HFD-induced diabetic mice was reduced by the knockdown of the miR-449a but increased by CEACAM1 knockdown (Fig. 5G). As shown in Fig. 5H-I, the increasing number of VECs in the STZ and HFD-induced diabetic mice were reduced by miR-449a inhibition, whereas the function of miR-449a inhibition was restored by CEACAM1 knockdown. Besides, the apoptosis of VECs was induced by miR-449a inhibition but reduced by CEACAM1 knockdown (Fig. 5J-K). The above results indicated that miR-449a silencing promoted plaque stability via targeting CEACAM1 in STZ and HFD-induced diabetic mice.

### MiR-449a accelerates inflammation and endothelial dysfunction via targeting CEACAM1 in HG-induced HUVECs

**Fig. 6:**
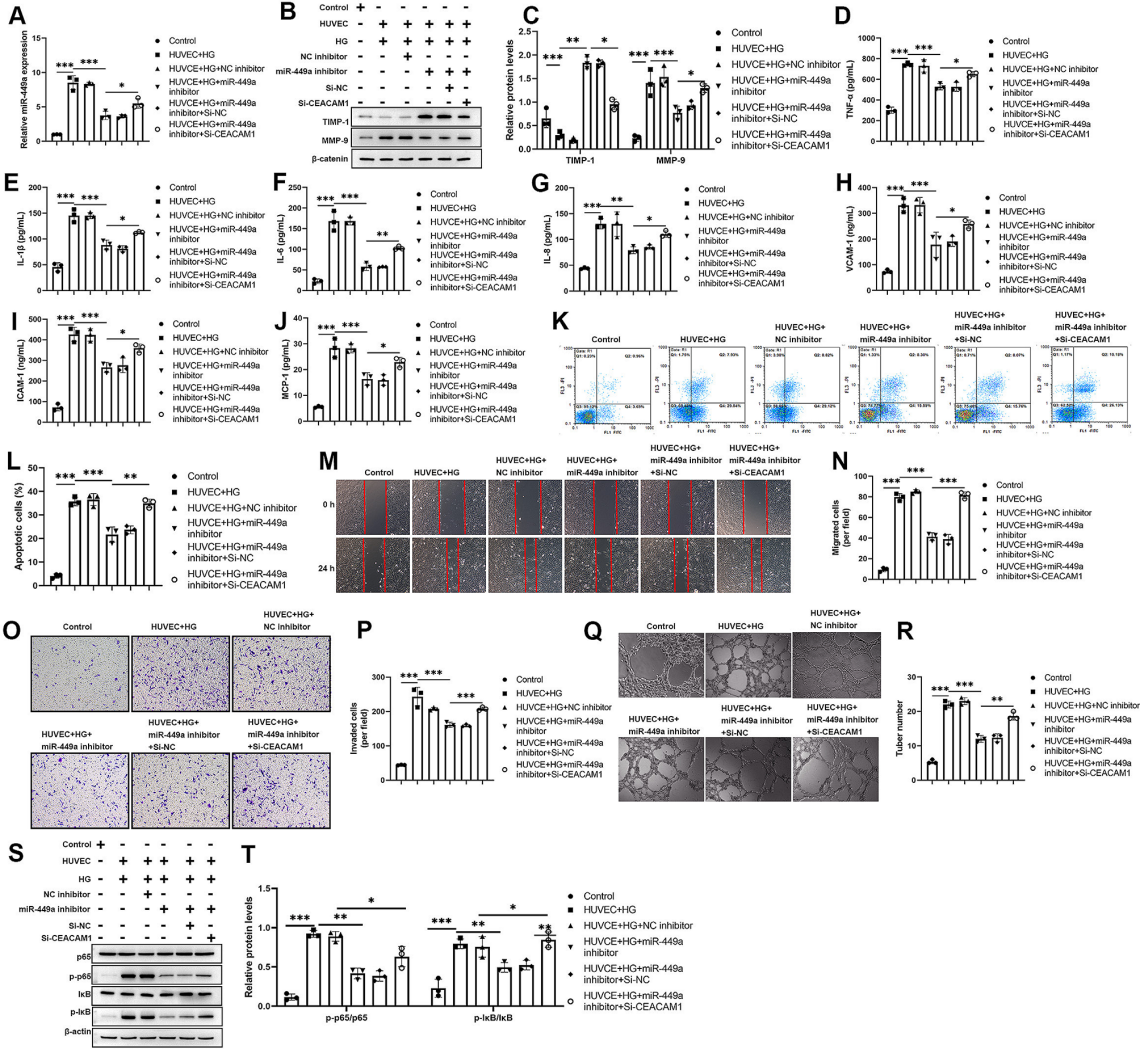
miR-449a accelerates inflammation and endothelial dysfunction via targeting CEACAM1 in HG-induced HUVECs. (**A**) qPCR analysis of expression of miR-449a after treatment with HG and transfection with miRNA inhibitors and small interfering RNAs in HUVECs. (**B**)-(**C**) Western blot analysis of the expression of TIMP-1 and MMP-9 after treatment with HG and transfection with miRNA inhibitors and small interfering RNAs in HUVECs. (**D**)-(**J**) ELISA analysis of the inflammation cytokines (TNF-α, IL-1β, IL-6, IL-8) and adhere molecules (VCAM-1, ICAM-1, MCP-1) after treatment with HG and transfection with miRNA inhibitors small interfering RNAs in HUVECs. (**K**)-(**L**) Annexin-V FITC/PI analysis of the cell apoptosis after treatment with HG and transfection with miRNA inhibitors and small interfering RNAs in HUVECs. (**M**)-(**P**) Wound healing and Transwell assays were used to detect cell migration and invasion after treatment with HG and transfection with miRNA inhibitors and small interfering RNAs in HUVECs. (**Q**)-(**R**) Tuber formation assay was utilized to analyze tuber formation ability after treatment with HG and transfection with miRNA inhibitors and small interfering RNAs in HUVECs. (**S**)-(**T**) Western blot analysis of the levels of p65 and IκB and the phosphorylation of p65 and IκB after treatment with HG and transfection with miRNA inhibitors and small interfering RNAs in HUVECs. Scatter plots represented values obtained from three individual experiments, Data are and the significances between those groups were obtained by the value (mean ± SD) were analyzed by one-way ANOVA. *P < 0.05; **P < 0.01; ***P < 0.001

Moreover, to further explore whether miR-449a affected endothelial dysfunction via targeting CEACAM1 in vitro. qPCR results indicated that HG promoted miR-449a upregulation, but miR-449a upregulation in HG-induced HUVECs was inhibited by miR-449a inhibition whereas enhanced by CEACAM1 knockdown (Fig. 6A). Western blotting results showed that HG increased MMP-9 levels but reduced TIMP-1 levels, the effects of HG on HUVECs were inhibited by miR-449a inhibition, and the effects of miR-449a inhibition were reversed by CEACAM1 knockdown (Fig. 6B-C). Moreover, the levels of TNF-α, IL-1β, IL-6, IL-8, VCAM-1, ICAM-1, and MCP-1 were increased by HG stimulating but reduced by miR-449a inhibition and enhanced by CEACAM1 knockdown (Fig. 6D-J). Moreover, the pro-apoptosis effect of HG on HUVECs was inhibited by miR-449a inhibition but enhanced by CEACAM1 knockdown (Fig. 6K-L). In addition, HG-induced cell migration and invasion were inhibited by miR-449a inhibition and increased by CEACAM1 knockdown (Fig. 6M-P). We also found that HG promoted tube formation, but miR-449a inhibition inhibited tube formation; however, CEACAM1 knockdown enhanced tube formation by partly neutralizing the effects of miR-449a inhibition (Fig. 6Q-R). The NF-κB pathway is proinflammation and is associated with inflammatory factors-induced angiogenesis [23]. Therefore, we further examined whether NF-κB pathway activation is involved in the HG-induced HUVECs. Western blotting revealed that HG activated the NF-κB pathway by promoting p65 and IκB phosphorylation, however, the HG effects on HUVECs were inhibited by miR-449a inhibition and enhanced by CEACAM1 knockdown (Fig. 6S-T). The above data indicated that miR-449a promoted inflammation and endothelial dysfunction by targeting CEACAM1 in HG-induced HUVECs.

## Discussion

The atherosclerotic macrovascular complication is a severe chronic inflammatory cardiovascular disease (CVD), and the physiological and pathological alternation during the initiation, development, and plaque disruption are modulated by inflammation [24]. Therefore, maintaining plaque stability and inhibiting inflammation are the strategies for alleviating AS to improve the life quality of DM patients eventually. In the present study, we found miR-449a and MMP-9 upregulated, whereas CEACAM1 was downregulated in DM-associated AS. Upregulation of CEACAM1 inhibited inflammation, promoted plaque stability in vivo, and suppressed endothelial dysfunction in vitro.

Much evidence has indicated that atherosclerotic lesion is a complex process involving multiple cell types that regulate plaque stability and inflammation. For example, macrophages contribute to endothelial lipid metabolism and produce pro-inflammatory mediators that cause arterial wall lesions [25]. Additionally, not only VSMCs exert a vital role in promoting fibrous caps, but also the non-VSMCs attend fibrous cap formation through endothelial-to-mesenchymal transition (EndoMT) or a macrophage-to-mesenchymal (MMT) [9]. CEACAM1 has been found to have abundant expression in epithelial, vessel endothelial, and hematopoietic cells, such as lymphocytes, macrophages, dendritic cells, and NK cells [26]. And CEACAM1 contributes to VECs migration, tuber formation, and vascular remodeling [27]. Our finding found that overexpression of CEACAM1 decreased the number of VECs in vivo and induced HUVEC migration, invasion, and tuber formation in vitro. Interestingly, CEACAM1 is also abundantly expressed in activated lymphocytes to promote IL-6 and IL-1β release [28]. In several cancers, CEACAM1 not only suppresses the inflammatory response and neoangiogenesis but also initiates extracellular matrix (ECM) remodeling during tumor development [29–31]. In vivo, we found that overexpression of CEACAM1 promotes collagen accumulation and increases the number of VSMCs but inhibits lipid deposition and the number of macrophages and VECs. Therefore, we deduced that CEACAM1 inhibited inflammation response and plaque instability.

miRNAs are a group of small non-coding RNAs that usually inhibit target gene expression by directly binding the 3’UTR of the mRNAs to attend to the biological processes of multiple diseases [32]. Increasing evidence has revealed that miRNAs modulate multiple critical biological processes correlated with AS [33], such as miR-342-5p, promoting HUVECs’ apoptosis to enhance atherosclerosis [34]. And miR-181b contributes to AS by targeting TIMP-3 and elastin [35]. MiR-155, derived from SMCs, induces endothelial injury and AS [36]. Our study found miR-449a upregulated in DM and HFD-induced AS and directly targeted to 3’UTR of CEACAM1. Moreover, miR-449a aggravated inflammation and plaque instability in DM and HFD-induced mice.

Commonly, the fibrous caps and immune cell infiltration are the essential characteristics of AS, which involve multiple cell types, including VECs, VSMCs, monocytes/macrophages, lymphocytes, and dendritic cells [37]. In addition, an imbalance of lipid metabolism is also a characteristic of AS, which causes excessing cholesterol-rich apolipoprotein B remnants and LDL accumulation in the endothelium and recruits monocytes to gather into the sub-endothelium [38]. Therefore, maintaining lipid metabolism balance is also a potential therapeutic strategy in the early stage of AS. Here, we provided evidence that downregulation of miR-449a decreased lipid deposition, inflammatory cytokines as well as the number of macrophages and VECs but increased collagen accumulation and the number of VSMCs via targeting CEACAM1 to inhibit the NF-κB pathway, which is a canonical pro-inflammatory pathway involves in atherosclerosis [39–41].

Although we demonstrated that the miR-449a/CEACAM1 axis played a dominant role in the development of the DM-associated AS, the present study still has a huge limitation. Increasing LDL, hyperglycemia, oxidative stress, and increased inflammation are the risk factors for AS [5]. Sustaining hyperglycemia is a leading cause of macro-and microvascular complications, and sustaining hyperglycemia is a common characteristic of T1DM and T2DM [42, 43]. While the role of metabolic abnormalities cannot be ignored, the pathways including advanced glycation end products, the renin-angiotensin system, oxidative stress, and increased expression of growth factors and cytokines were shown to contribute a causal role in the formation of atherosclerotic plaques and account for the increased risk of macrovascular complications [44]. Streptozotocin (STZ) is a broad-spectrum antibiotic derived from Streptomyces [45]. After injection with a large dose or with multiple injections in small dose, it directly damages pancreatic islet B-cells, destroys B-cells by activating autoimmunity, increases the body’s oxidative capacity to damage pancreatic endocrine cells, and ultimately decreases insulin secretion, leading to increased blood glucose levels [46, 47]. Here, we constructed the STZ-induced mice to verify the regulatory mechanism for DM-associated AS. STZ causes the destruction of the pancreatic islet in diabetes mice [48], which is more similar to the insulin-dependent T1DM [49]. While the subsequent HFD influenced the insulin secretion but not induced insulin resistant. This results in a limitation of the model to fully mimic the complicated pathology development in DM-associated AS. Although previous studies have indicated that genetically obese rodent model db/db mice are more similar to T2DM [50, 51], it is recognized, that STZ-induced ApoE^−/−^ mice have shown more stable and recognized spontaneous atherosclerotic plaques [52, 53]. Moreover, another limitation was that we constructed the HG-induced HUVECs to examine the function and mechanism of the miR-449a/CEACAM1 axis in vitro. There are not only VECs but also VSMCs and some lymphocytes involved in the processing of atherosclerosis. However, in the present study, the limitations will not affect the results and conclusion that miR-449a promotes atherosclerotic plaque rupture, inflammation, and endothelial dysfunction by interacting with CEACAM1.

## Conclusion

In conclusion, we unveiled that miR-449a plays a vital role in promoting plaque friability and proinflammation via targeting CEACAM1 and NF-κB pathways. Therefore, our findings might supply efficiently potential therapeutic targets for AS to improve the current status of treatment DM.

### Electronic supplementary material

Below is the link to the electronic supplementary material.

Supplementary Material 1

Supplementary Material 2

Supplementary Material 3

## Data Availability

The datasets used and/or analyzed during the current study are available from the corresponding author upon reasonable request.

## References

[1] Chatterjee S, Khunti K, Davies MJ (2017). Type 2 diabetes. Lancet.

[2] Beckman JA, Creager MA, Libby P (2002). Diabetes and atherosclerosis: epidemiology, pathophysiology, and management. JAMA.

[3] Orchard TJ (2006). Type 1 diabetes and coronary artery disease. Diabetes Care.

[4] Haas AV, McDonnell ME (2018). Pathogenesis of Cardiovascular Disease in Diabetes. Endocrinol Metab Clin North Am.

[5] Poznyak A (2020). The diabetes Mellitus-Atherosclerosis connection: the role of lipid and glucose metabolism and chronic inflammation. Int J Mol Sci.

[6] Wu Y (2014). Risk factors contributing to type 2 diabetes and recent advances in the treatment and prevention. Int J Med Sci.

[7] Tuñón J (2018). Interplay between hypercholesterolaemia and inflammation in atherosclerosis: translating experimental targets into clinical practice. Eur J Prev Cardiol.

[8] Falk E (2006). Pathogenesis of atherosclerosis. J Am Coll Cardiol.

[9] Newman AAC (2021). Multiple cell types contribute to the atherosclerotic lesion fibrous cap by PDGFRβ and bioenergetic mechanisms. Nat Metab.

[10] Zhang Y (2022). The management correlation between metabolic index, cardiovascular health, and diabetes combined with cardiovascular disease. Front Endocrinol (Lausanne).

[11] Nouvion AL (2010). CEACAM1: a key regulator of vascular permeability. J Cell Sci.

[12] Kleefeldt F (2019). Aging-related carcinoembryonic antigen-related cell adhesion molecule 1 signaling promotes vascular dysfunction. Aging Cell.

[13] Russo L (2018). Liver-specific rescuing of CEACAM1 reverses endothelial and cardiovascular abnormalities in male mice with null deletion of Ceacam1 gene. Mol Metab.

[14] Rueckschloss U, Kuerten S, Ergün S (2016). The role of CEA-related cell adhesion molecule-1 (CEACAM1) in vascular homeostasis. Histochem Cell Biol.

[15] Yu J (2020). CEACAM1 inhibited IκB-α/NF-κB Signal Pathway Via Targeting MMP-9/TIMP-1 Axis in Diabetic Atherosclerosis. J Cardiovasc Pharmacol.

[16] Lu Y (2018). Impact of miRNA in atherosclerosis. Arterioscler Thromb Vasc Biol.

[17] Feinberg MW, Moore KJ (2016). MicroRNA Regul Atherosclerosis Circ Res.

[18] Weng C, Nguyen T, Shively JE (2016). miRNA-342 regulates CEACAM1-induced lumen formation in a three-dimensional model of mammary gland morphogenesis. J Biol Chem.

[19] Zhao C (2020). Up-regulation of microRNA-30b/30d cluster represses hepatocyte apoptosis in mice with fulminant hepatic failure by inhibiting CEACAM1. IUBMB Life.

[20] Jiang L (2019). miR-449a induces EndMT, promotes the development of atherosclerosis by targeting the interaction between AdipoR2 and E-cadherin in lipid rafts. Biomed Pharmacother.

[21] Chen LB (2017). A single nucleotide polymorphism located in microRNA-499a causes loss of function resulting in increased expression of osbpl1a and reduced serum HDL level. Oncol Rep.

[22] Han WM (2020). Acacetin protects against high glucose-Induced endothelial cells Injury by preserving mitochondrial function via activating Sirt1/Sirt3/AMPK signals. Front Pharmacol.

[23] Kumar A (2016). Daphnetin inhibits TNF-α and VEGF-induced angiogenesis through inhibition of the IKKs/IκBα/NF-κB, Src/FAK/ERK1/2 and akt signalling pathways. Clin Exp Pharmacol Physiol.

[24] Zhu Y (2018). Research Progress on the relationship between atherosclerosis and inflammation. Biomolecules.

[25] Qiao XR (2020). MiR-210-3p attenuates lipid accumulation and inflammation in atherosclerosis by repressing IGF2. Biosci Biotechnol Biochem.

[26] Gray-Owen SD, Blumberg RS (2006). CEACAM1: contact-dependent control of immunity. Nat Rev Immunol.

[27] MacManiman JD (2014). Human cytomegalovirus-encoded pUL7 is a novel CEACAM1-like molecule responsible for promotion of angiogenesis. mBio.

[28] Zhang Z (2019). CEACAM1 regulates the IL-6 mediated fever response to LPS through the RP105 receptor in murine monocytes. BMC Immunol.

[29] Samineni S, Zhang Z, Shively JE (2013). Carcinoembryonic antigen-related cell adhesion molecule 1 negatively regulates granulocyte colony-stimulating factor production by breast tumor-associated macrophages that mediate tumor angiogenesis. Int J Cancer.

[30] Shi JF (2014). Expression of carcinoembryonic antigen-related cell adhesion molecule 1(CEACAM1) and its correlation with angiogenesis in gastric cancer. Pathol Res Pract.

[31] Gerstel D (2011). CEACAM1 creates a pro-angiogenic tumor microenvironment that supports tumor vessel maturation. Oncogene.

[32] Rupaimoole R, Slack FJ (2017). MicroRNA therapeutics: towards a new era for the management of cancer and other diseases. Nat Rev Drug Discov.

[33] Churov A (2019). MicroRNAs as potential biomarkers in atherosclerosis. Int J Mol Sci.

[34] Xing X (2020). Adipose-derived mesenchymal stem cells-derived exosome-mediated microRNA-342-5p protects endothelial cells against atherosclerosis. Aging.

[35] Di Gregoli K (2017). MicroRNA-181b controls atherosclerosis and Aneurysms through Regulation of TIMP-3 and Elastin. Circ Res.

[36] Zheng B (2017). Exosome-mediated miR-155 transfer from smooth muscle cells to endothelial cells induces endothelial Injury and promotes atherosclerosis. Mol Ther.

[37] Bobryshev YV (2008). Matrix vesicles in the fibrous cap of atherosclerotic plaque: possible contribution to plaque rupture. J Cell Mol Med.

[38] Remmerie A, Scott CL (2018). Macrophages and lipid metabolism. Cell Immunol.

[39] Mitchell S, Vargas J, Hoffmann A (2016). Signaling via the NFκB system. Wiley Interdiscip Rev Syst Biol Med.

[40] Arya P, Bhandari U (2020). Involvement of the toll-like receptors-2/nuclear factor-kappa B signaling pathway in atherosclerosis induced by high-fat diet and zymosan A in C57BL/6 mice. Indian J Pharmacol.

[41] Zhou ZB (2018). Lysophosphatidic acid promotes expression and activation of Matrix Metalloproteinase 9 (MMP9) in THP-1 cells via toll-like receptor 4/Nuclear Factor-κB (TLR4/NF-κB) signaling pathway. Med Sci Monit.

[42] Nagareddy PR (2013). Hyperglycemia promotes myelopoiesis and impairs the resolution of atherosclerosis. Cell Metab.

[43] Juutilainen A (2008). Similarity of the impact of type 1 and type 2 diabetes on cardiovascular mortality in middle-aged subjects. Diabetes Care.

[44] Calkin AC, Allen TJ (2006). Diabetes mellitus-associated atherosclerosis: mechanisms involved and potential for pharmacological invention. Am J Cardiovasc Drugs.

[45] Karunanayake EH, Hearse DJ, Mellows G (1976). Streptozotocin: its excretion and metabolism in the rat. Diabetologia.

[46] Like AA, Rossini AA (1976). Streptozotocin-induced pancreatic insulitis: new model of diabetes mellitus. Science.

[47] Papaccio G (1991). Early macrophage infiltration in mice treated with low-dose streptozocin decreases islet superoxide dismutase levels: prevention by silica pretreatment. Acta Anat (Basel).

[48] Utsugi T (1996). Major histocompatibility complex class I-restricted infiltration and destruction of pancreatic islets by NOD mouse-derived beta-cell cytotoxic CD8 + T-cell clones in vivo. Diabetes.

[49] Li W, Huang E, Gao S (2017). Type 1 diabetes Mellitus and cognitive impairments: a systematic review. J Alzheimers Dis.

[50] Okada-Iwabu M (2013). A small-molecule AdipoR agonist for type 2 diabetes and short life in obesity. Nature.

[51] Michurina SV (2020). Apoptosis in the liver of male db/db mice during the development of obesity and type 2 diabetes. Vavilovskii Zhurnal Genet Selektsii.

[52] Liang WJ (2018). AMPKα inactivation destabilizes atherosclerotic plaque in streptozotocin-induced diabetic mice through AP-2α/miRNA-124 axis. J Mol Med (Berl).

[53] Kako Y (1999). Streptozotocin-induced diabetes in human apolipoprotein B transgenic mice. Effects on lipoproteins and atherosclerosis. J Lipid Res.

