# Supplementary figures and images for: miR-449a disturbs atherosclerotic plaque stability in streptozotocin and high-fat diet-induced diabetic mice by targeting CEACAM1
Source: Diabetol Metab Syndr. 2024 May 8;16:98. doi: 10.1186/s13098-024-01322-y (PMC11077876; doi:10.1186/s13098-024-01322-y)

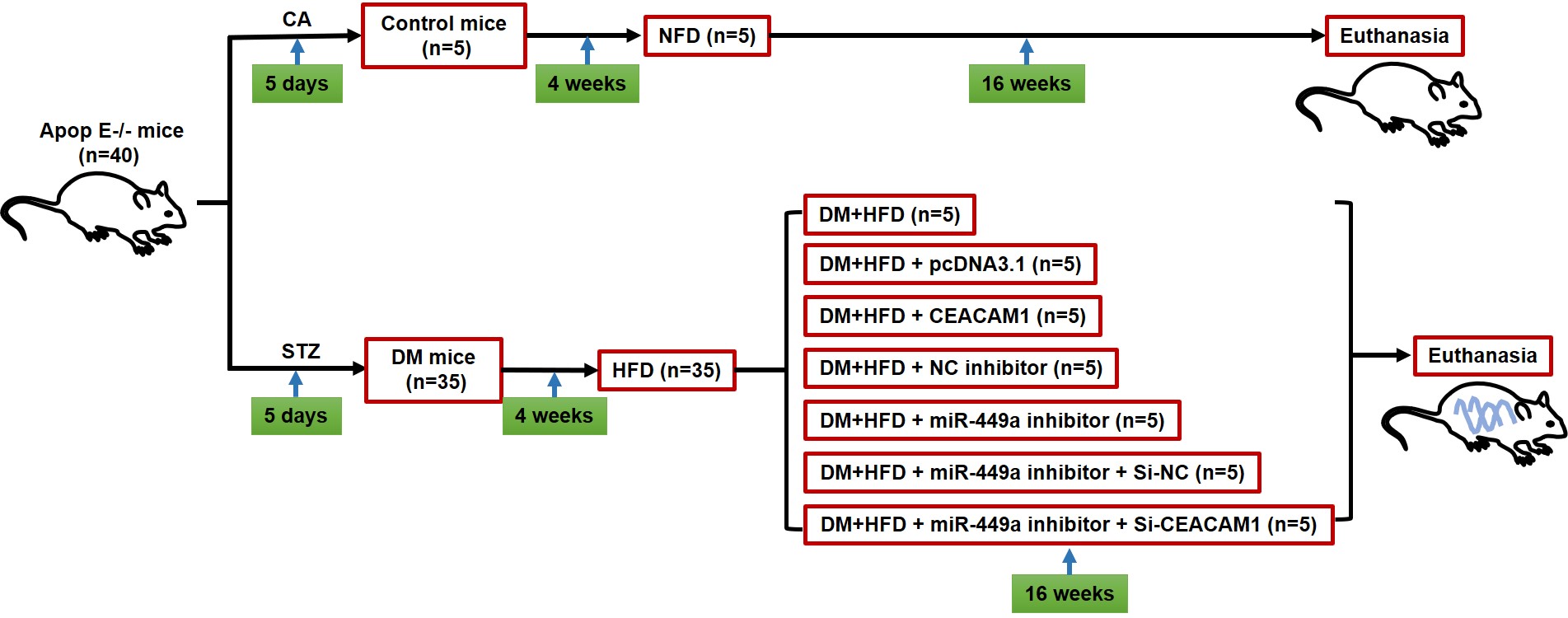

Supplement: Supplementary file 1 — Supplementary Material 1 [file 13098_2024_1322_MOESM1_ESM.jpg]

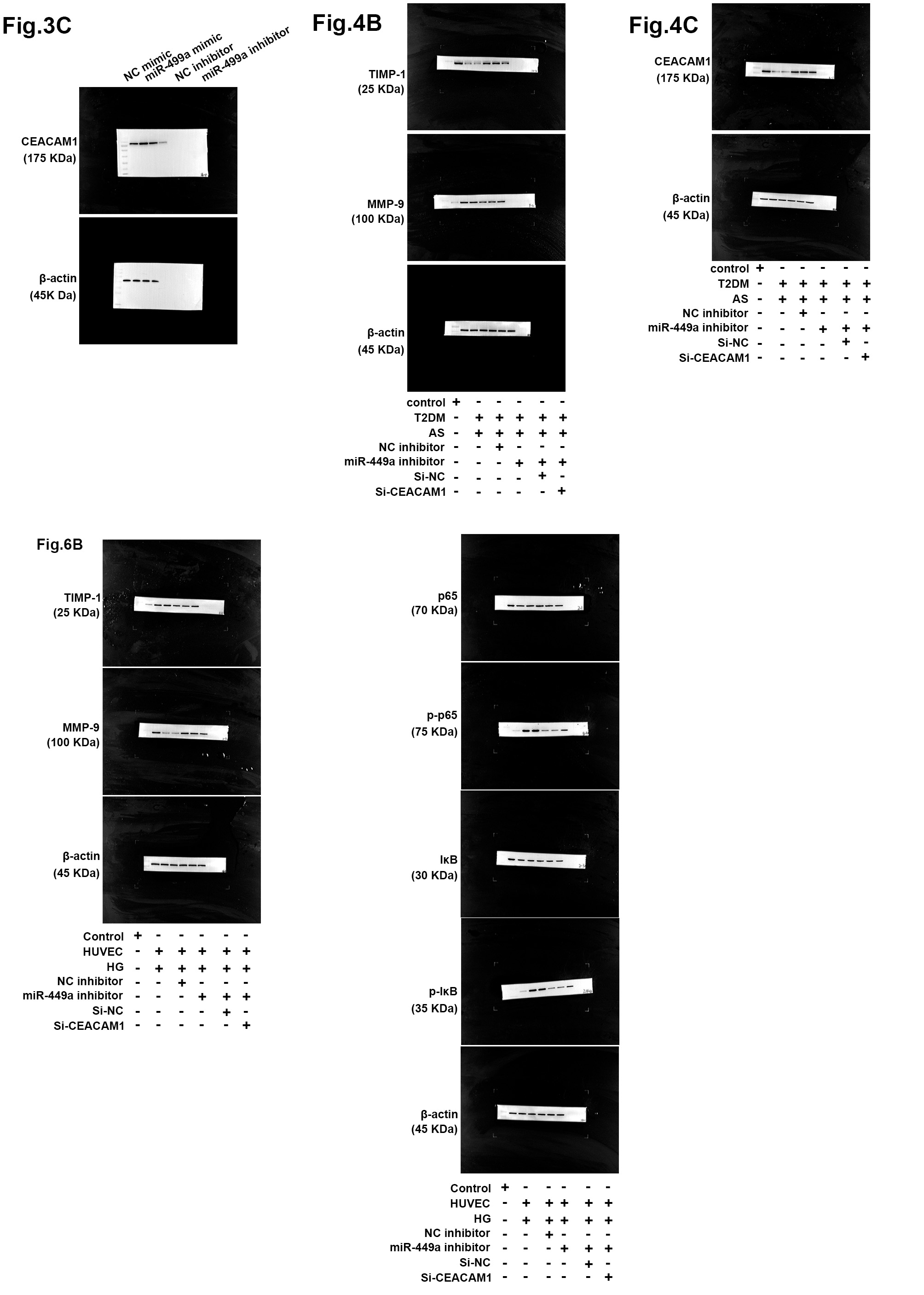

Supplement: Supplementary file 2 — Supplementary Material 2 [file 13098_2024_1322_MOESM2_ESM.jpg]
