# Supplementary material for: miR-449a disturbs atherosclerotic plaque stability in streptozotocin and high-fat diet-induced diabetic mice by targeting CEACAM1
Source: Diabetol Metab Syndr. 2024 May 8;16:98. doi: 10.1186/s13098-024-01322-y (PMC11077876; doi:10.1186/s13098-024-01322-y)
Supplement: Supplementary file 3 — Supplementary Material 3 [file 13098_2024_1322_MOESM3_ESM.pdf]

Supplementary Figure 1

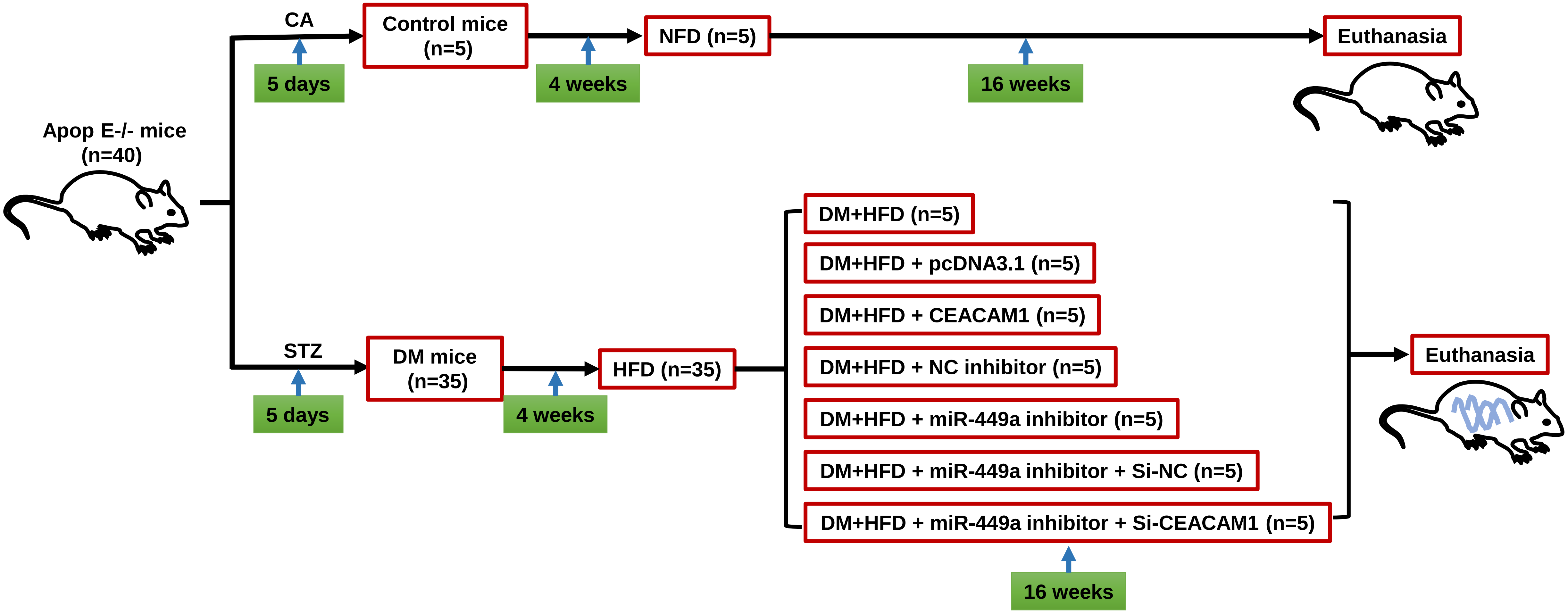

Supplementary Figure 3D

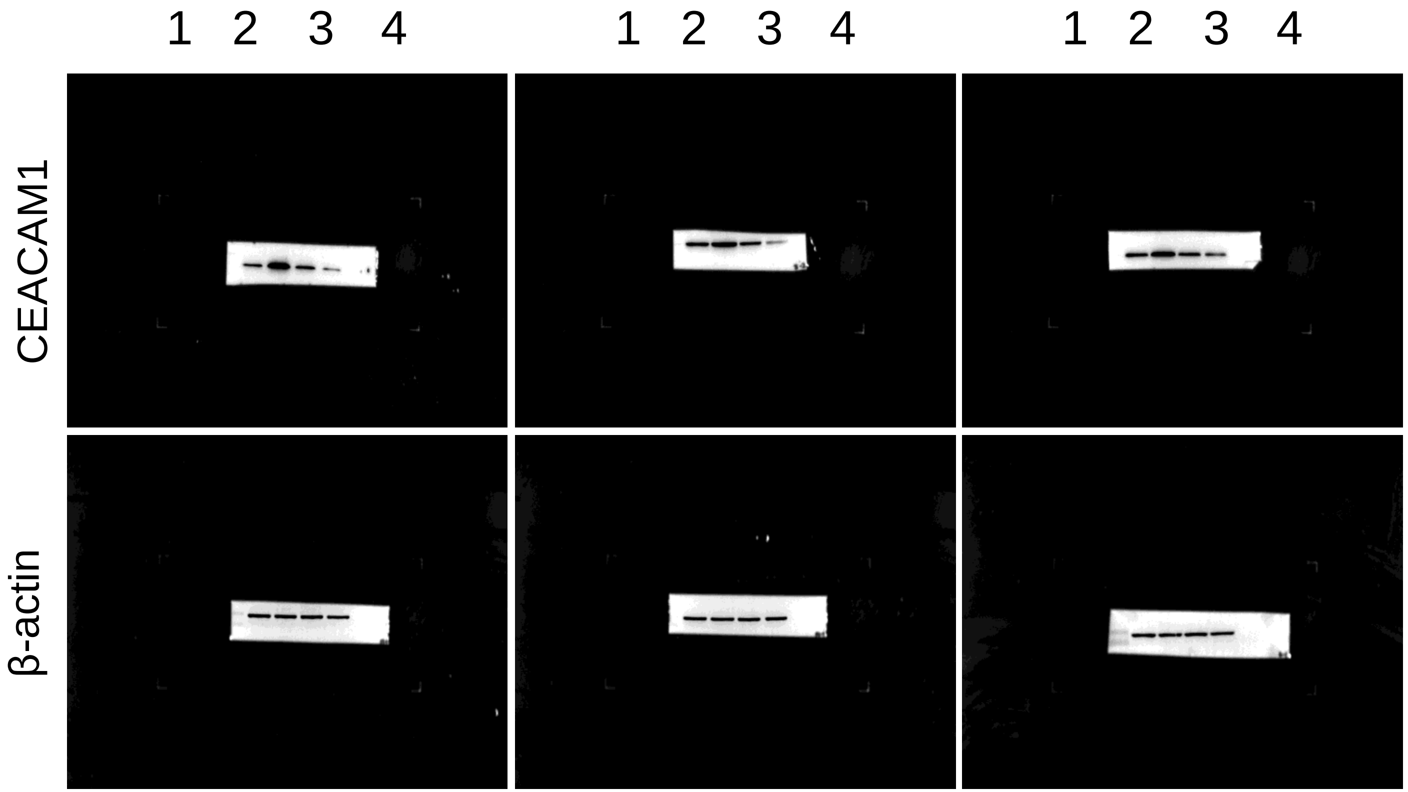

Groups
1 NC inhibitor
2 miR-449a inhibitor
3 NC mimic
4 miR-449a mimic

Supplementary Figure 4B

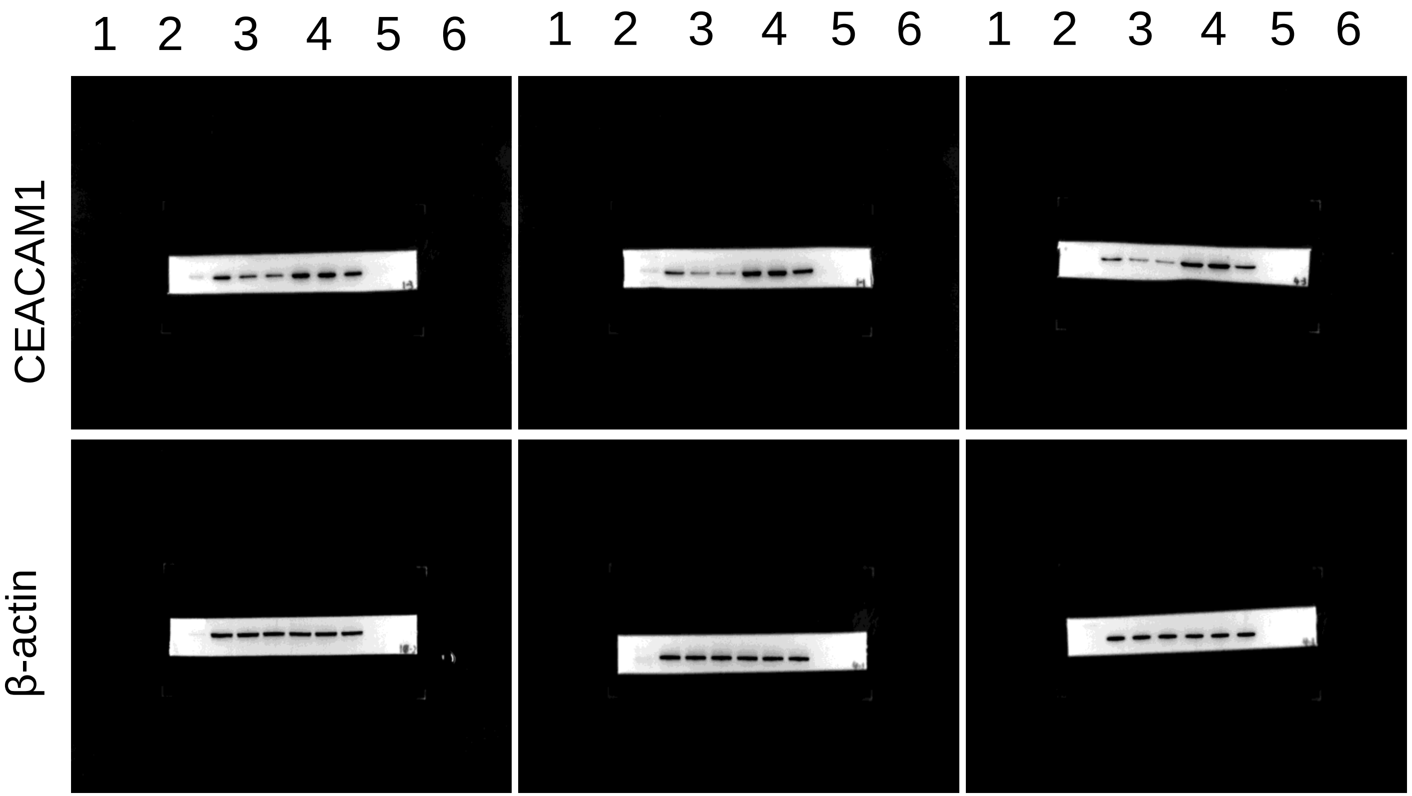

Groups
1 Control
2 DM+HFD
3 DM+HFD+NC inhibitor
4 DM+HFD+miR-449a inhibitor
5 DM+HFD+miR-449a inhibitor+si-NC
6 DM+HFD+miR-449a inhibitor+si-CEACAM1

Supplementary Figure 4D

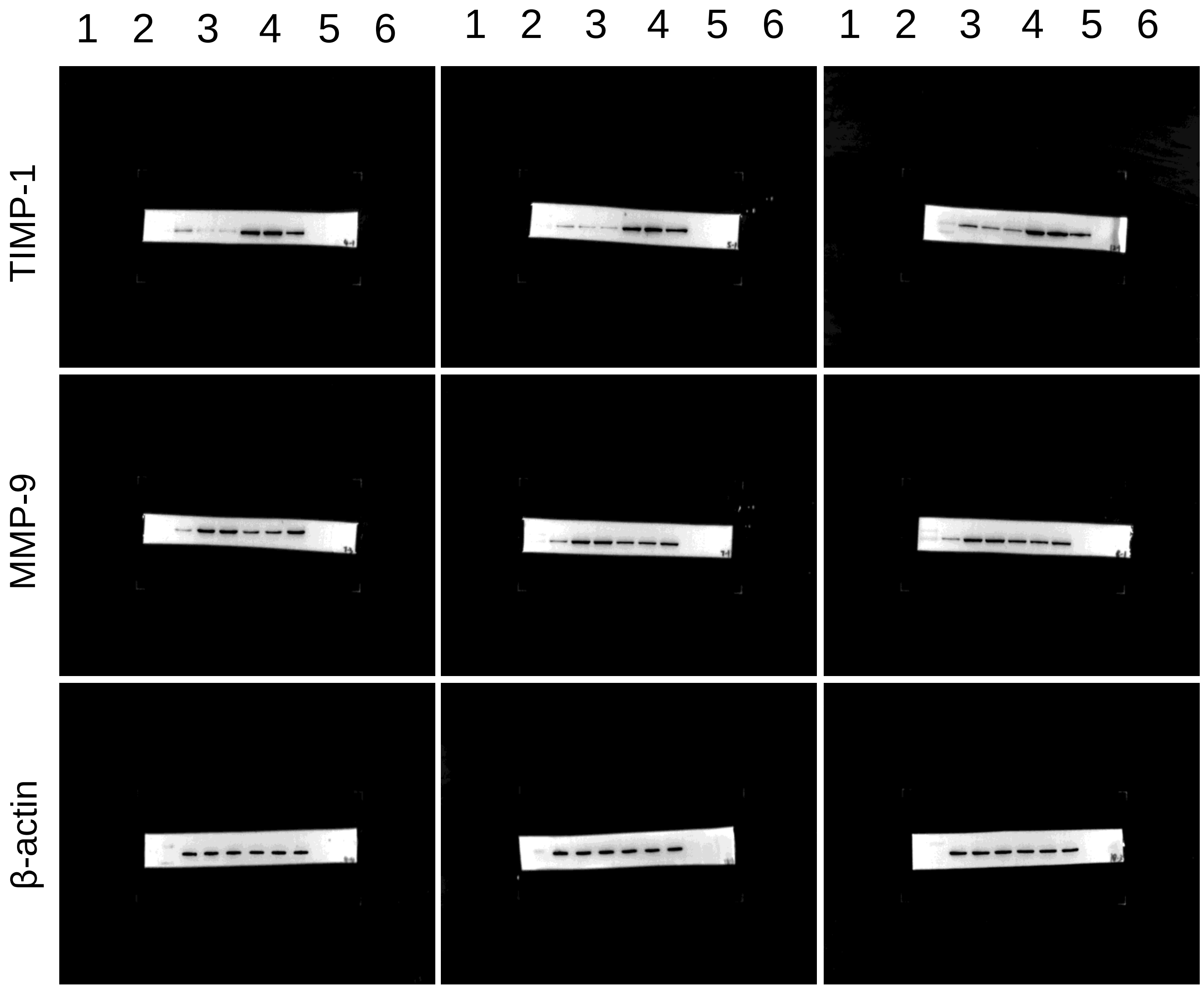

Groups
1 Control
2 DM+HFD
3 DM+HFD+NC inhibitor
4 DM+HFD+miR-449a inhibitor
5 DM+HFD+miR-449a inhibitor+si-NC
6 DM+HFD+miR-449a inhibitor+si-CEACAM1

Supplementary Figure 6B

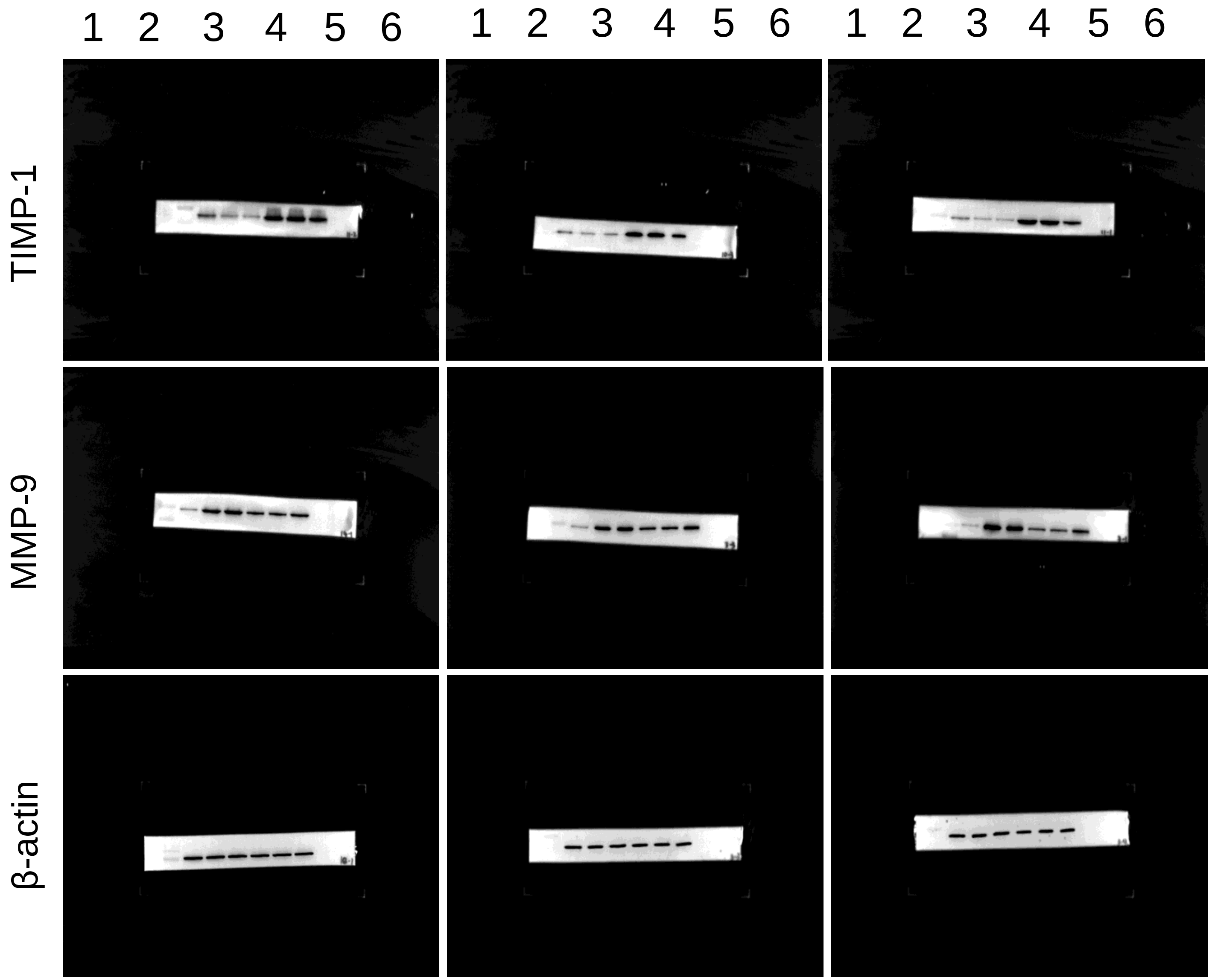

Groups
1 Control
2 HUVEC+HG
3 HUVEC+HG +NC inhibitor
4 HUVEC+HG +miR-449a inhibitor
5 HUVEC+HG +miR-449a inhibitor+si-NC
6 HUVEC+HG +miR-449a inhibitor+si-CEACAM1

Supplementary Figure 6S

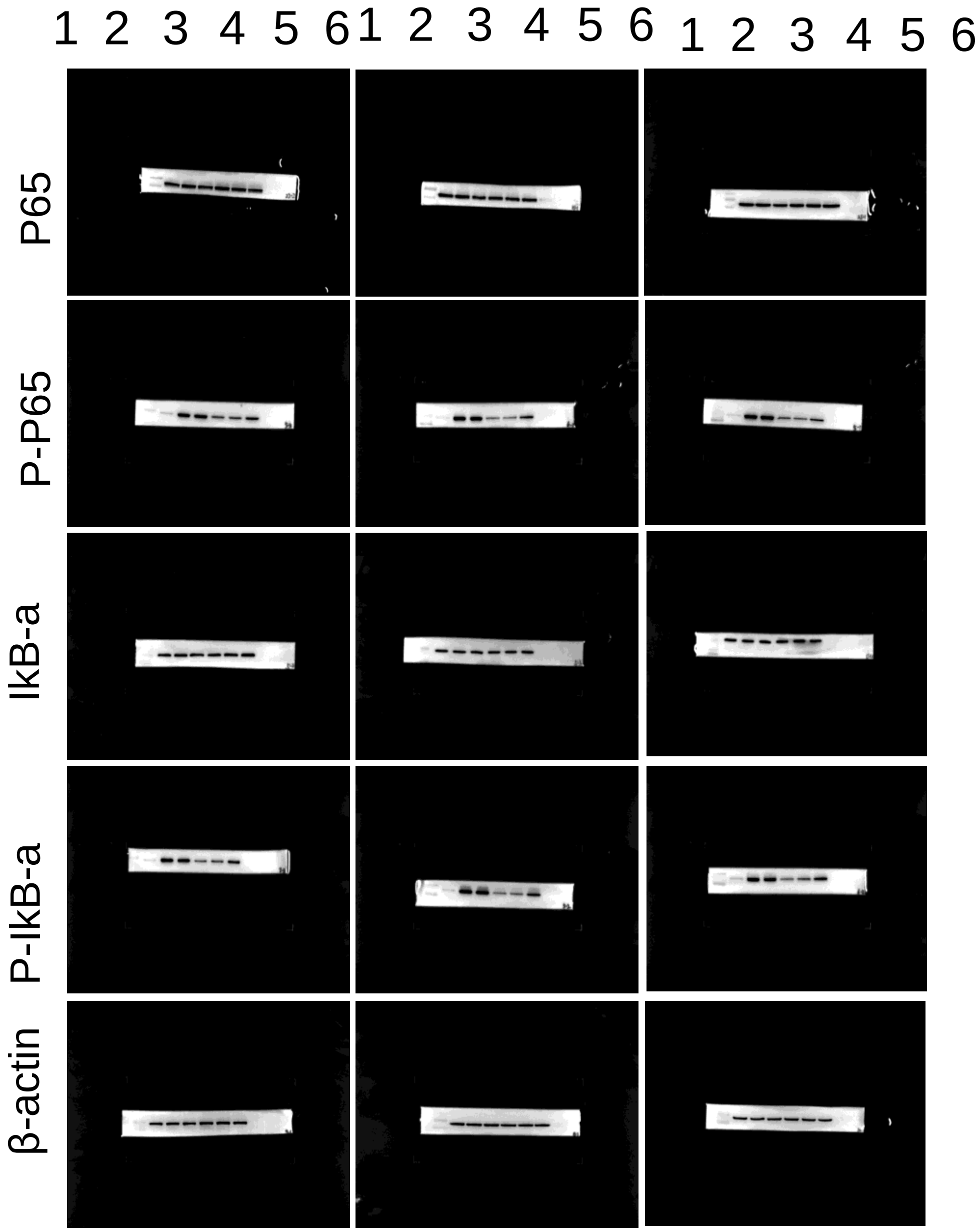

Groups
1 Control
2 HUVEC+HG
3 HUVEC+HG +NC inhibitor
4 HUVEC+HG +miR-449a inhibitor
5 HUVEC+HG +miR-449a inhibitor+si-NC
6 HUVEC+HG +miR-449a inhibitor+si-CEACAM1
